# An investigation on chemical/mineral compositions, ruminal microbial fermentation, and feeding value of some leaves as alternative forages for finishing goats during the dry season

**DOI:** 10.1186/s13568-021-01238-0

**Published:** 2021-05-27

**Authors:** Mohsen Kazemi

**Affiliations:** Department of Animal Science, Faculty of Agriculture and Animal Science, University of Torbat-e Jam, Torbat-e Jam, Iran

**Keywords:** Feed, Goat, Leaves, Nutritional value, Growth performance

## Abstract

Finding new fodder resources with moderate to high nutritional value that are cheaper and available is one of the most challenges in livestock industries. Hence, the nutritive value of some tree leaves (quince, pear, olive, mirabelle plum, greengage, sour cherry, and persimmon) was investigated by different laboratories and in vitro methods. Also, partial substitution of alfalfa and corn silage (50%) with these leaves was investigated using forty-eight goats in a randomized complete block design in vivo. Highest total phenol and tannin contents were obtained in quince (*p* < 0.001). Greengage (146.37 g/kg DM) and persimmon (136.96 g/kg DM) exhibited the highest crude protein, respectively (*p* < 0.001). Calcium content (19.82 g/kg DM) was highest in persimmon leaves (*p* < 0.001). Greengage (66.07 mmol/L) and mirabelle plum (65.58 mmol/L) produced more total volatile fatty acids in the culture medium, respectively (*p* < 0.001). Potential gas production ranged from 39.65 mL for pear to 55.32 mL mirabelle plum. Sour cherry had the highest acid–base buffering capacity (183.73 mEq × 10^–3^, *p* < 0.001). Highest dry matter intake (1087 g/day) and crude protein digestibility (70.80 %) were observed in diets containing greengage (*p* < 0.001). Total antioxidant capacity of serum increased when olive, quince, and persimmon were considered in goats feeding (*p* < 0.001). Although all studied leaves can be fed in diets of goats without deleterious effects on performance, serum, and hematology parameters, in vivo and in vitro results indicated that greengage in terms of nutritive value was superior to other studied leaves.

## Introduction

Due to the growing population, the global demand for animal proteins is increasing. Also, the supply of animal feeds is one of the major costs associated with livestock industries. Therefore, searching for alternative feed resources that are palatable and low-cost, and can meet the nutritional requirements of small ruminants is one of the major goals for livestock farming (Abegunde and Akinsoyinu 2011). Foliage leaves are major feed resources for small ruminant production in arid and semi-arid regions of Iran, especially in smallholder farms. Also, in these areas, the leaves of some trees are rich in nutrients and can be grazed in the fall season by small ruminants when they fall to the ground. It is reported that tree leaves contain moderate levels of nitrogen, minerals, and vitamins (Patra 2009). Also, it is reported that leaves of mulberry trees as a protein resource could be used in sheep diets containing ammoniated rice straw and rapeseed meal (Liu et al. 2001). The substitution of conventional ingredients by tree leaves will make diets cheaper rather than commercial feeds (Ondiek et al. 2000). Although foliage leaves have been extensively used in small ruminant feeding, their excessive consumption due to the secondary compounds (such as phenol and tannin) can be harmful to the small ruminants (Salem 2005; Salem et al. 2006). However, it is well known that moderate levels of tannins in the ration (3–4% of DM tannins) can have beneficial effects on the availability of protein for ruminants (Min and Solaiman 2018). Quince is a plant belonging to *Rosaceae* whose medicinal characteristics as a result of phenolic compounds are reported extensively (Ashraf et al. 2016). Pear with a diverse range of phenolic compounds is one of the first broadly consumed fruits within the world and utilized as a traditional cure in Asia due to their antitussive, anti-inflammatory, and diuretic exercises (Jung et al. 2004; Cui et al. 2005; Fischer et al. 2007). Olive leaves as agricultural residues are reported to be rich in total phenol (63.5 g/kg DM) and lignin (109 g/kg DM) contents (Abbeddou et al. 2011). The Mirabelle plum from the *Rosaceae* family is principally known for high dietary fiber, sorbitol, phenolic compounds (commonly as chlorogenic and neochlorogenic acids), potassium, and boron (Stacewicz-Sapuntzakis et al. 2001), organoleptic and nutritional characteristics (Ioannou et al. 2011). A suitable level of minerals is reported for two varieties of greengage cultivar (*Rosaceae* family) in Spain (Reig et al. 2018). Sour cherry with high polyphenol and flavonoid contents belongs to the *Rosaceae* family whose antioxidant capacity of leaves is proven (Nowak et al. 2016). Persimmon with potential antioxidant activity is a plant of the *Ebenaceae* family, whose leaves have different therapeutic effects on the cardiovascular system, blood pressure, myocardial apoptosis, immune system, etc. (Xie et al. 2015). Lack of general information on the nutritive value of alternative forage resources can lead to unbalanced diets, low livestock growth and animal protein production, and subsequently low income for animal husbandries. Hence, different laboratories, in vitro, and in vivo methods were designed for determining and comparing the nutritive value of some tree leaves (quince, pear, olive, mirabelle plum, greengage, sour cherry, and persimmon) available in Iran and other countries.

## Material and methods

### Area collection and leaves samples

Different orchards located in Kashmar city were randomly selected for leaf sampling [i.e., quince (*Cydonia oblonga* Mill.), pear (*Pyrus communis* L.), olive (*Olea europaea* L.), mirabelle plum (*Prunus domestica* L.), greengage (*Prunus domestica* L.), sour cherry (*Prunus cerasus* L.), and persimmon (*Diospyros kaki* L.)]. Kashmar (35°14′N and 58°27′E, 206 mm average rainfall, and an average temperature of 17.7 °C) with semi-arid weather is located in the north east of Iran, Khorasan Razavi province. The leaves were randomly picked by hand from different orchards when ~ 40% of the leaves had fallen and 80% had turned yellow. For the in vitro trial, the leaves of 20 different trees were randomly collected, mixed and a 5 kg subsample was transferred to the laboratory for more analysis. The collected leaves from different orchards were left on the ground to be dried (with 90% DM) and then preserved in a roofed barn until the start of the in vivo experiment.

### Laboratory analysis

The samples of fresh leaves were moved to an air-forced oven (Behdad Co., Iran) (method no. 930.15) for dry matter (DM) determination (AOAC 2005). The methods recommended by AOAC (2005) were used for the determination of ash (method no. 942.05), ether extract (EE, method no. 991.36), and crude protein (CP, Kjeldahl, N × 6.25, method no. 954.01) contents. The concentrations of acid detergent fiber (ADF), acid detergent lignin (ADL), and neutral detergent fiber (NDF) were determined by procedures of Ankom technology (2005,2006a; b) with reagents described by Van Soest et al. (1991). The concentrations of total phenolic, tannins, and non-tannin phenolic (NTP) were determined according to protocol of Makkar (2003b). The procedure of Jasaitis et al. (1987) was employed to determine the buffering capacity and pH of the samples. An atomic absorption spectrophotometer (SavantAA, GBC, Australia) was employed to determine mineral contents (calcium, phosphorus, potassium, sodium, magnesium, manganese, cobalt, iron, zinc, and copper). Phosphorus was determined by a UV–visible spectrophotometer (Photonix-Ar-2017, Iran) using the molybdovanadate method.

### In vitro rumen fermentation

The method of Menke and Steingass (1988) was employed for the run of in vitro gas test. Rumen fluid was collected from three fistulated Sistani male goats, strained through four layers of cheesecloth, and kept in a warm water bath at 39 °C. The diet of animals was prepared based on NRC (2007) at the maintenance level (60:40 forage-to-concentrate ratio). About 200 mg of each leaf sample (1 mm screen, Arthur H. Thomas, Philadelphia, PA, USA) was placed into a 100 mL glass syringe with four replicates. Rumen fluid and artificial saliva (30 mL) were mixed in a 1:2 ratio, added to each syringe, and incubated in a water bath at 39 °C for 3, 6, 9, 12, 24, 48, 72, and 96 h. The respective gas production was recorded at the each of mentioned times. Four syringes without leaf samples in each run were also considered as blank. The gas test was replicated in two runs.

The pH of the culture medium was determined immediately with a pH meter (Hana, Model HI 2210-01, USA) following the filtration of each syringe content. After 24 h incubation, the content of each syringe was strained using a Buchner funnel equipped with a polyester cloth (45-micron pore size, Kazemi and Ghasemi Bezdi 2021). The strained content was moved into the pre-weighed crucible, washed with the neutral detergent solution, and dried in a forced-air oven at 60 °C (Makkar 2010) for 48 h. The method of Makkar (2010) was used for true DM and organic matter digestibility (TDMD and TOMD), and partitioning factor (PF) determination. The amount of 10 mL of the filtered solution was mixed with 10 mL of 0.2 N HCl and stored in a – 18 °C freezer until ammonia nitrogen (NH_3_-N) analysis. The concentration of NH_3_-N was determined by the Kjeldahl method (Komolong et al. 2001). The protocol described by Getachew et al. (2004) was used for sampling of total volatile fatty acids (VFA). The analysis of VFA was done by gas chromatography equipped (YL6100 GC; Young Lin Instrument, Anyang, South Korea) with a 50 m silica-fused (0.32 mm ID) column (CP-Wax Chrompack Capillary Column, Varian, Palo Alto, CA, USA). Internal standard and carrier gas were crotonic acid (trans-2-butenoic acid) and helium, respectively. Initial and final oven temperatures were 55 and 195 °C, respectively. A temperature of 250 °C was set for the detector and injector.

### Treatments and animal experiment

Forty-eight Sistani male goats (19 ± 1.5 kg, 7-month-old) were allocated to eight treatment (n = 6 per treatment). Treatments were (1) control (basal diet containing alfalfa and corn silage); (2) Quince (50% alfalfa and 50% corn silage were substituted with quince leaves); (3) Pear (50% alfalfa and 50% corn silage were substituted with pear leaves); (4) Olive (50% alfalfa and 50% corn silage were substituted with olive leaves); (5) Mirabelle Plum (50% alfalfa and 50% corn silage were substituted with mirabelle plum); (6) Greengage (50% of alfalfa and 50% corn silage were substituted with greengage leaves); (7) Sour cherry (50% of alfalfa and 50% corn silage were substituted with sour cherry leaves); (8) Persimmon (50% of alfalfa and 50% corn silage were substituted with persimmon leaves). This project was conducted in a sheep husbandry farm in Kashmar, Khorasan Razavi, Iran. Before the experiment started, goats were dewormed with albendazole. Each animal was kept in a 2 m × 2 m individual pen with free access to clean water. A 14-day adaptation was considered before the treatments are applied. Diets were formulated based on NRC instructions (2007) with a forage to concentrate ratio of 40:60 (DM basis). Feed was offered to animals twice a day at 07:00 and 19:00 h. After overnight fasting, goats were weighted before and every 30 days after the trial for monitor body weight change. The experiment lasted for 90 days. Feed intake and refusal were recorded throughout the trial. The animals were allowed to acclimatize to the fecal bags for 3 days. On the 83rd day of the experiment, a 7-day period was considered for the digestion trial. Total fecal was collected into the fecal bags. These bags were emptied and weighted two times a day. Sampling from total fecal, feed, and refusal was done for DM and chemical analysis. Blood samples were taken 3 h after morning feeding via jugular vein and the mean of two consecutive days (89 and 90) was considered for final statistical analysis. The collected blood samples were divided into two tubes, one containing ethylenediaminetetraacetic acid (EDTA) as an anticoagulant agent for hematology assessment and the other was anticoagulant free for biochemical assay. The concentration of hemoglobin (Hb), white blood cells (WBC), red blood cells (RBC), packed cell volume (PCV), mean corpuscular hemoglobin (MCH), mean corpuscular volume (MCV), and mean corpuscular hemoglobin (MCHC) were determined using an automated hematology analyzer (CellTac α, MEK-6450, Nihon Kohden, Japan). The malondialdehyde (MDA) concentration was determined as thiobarbituric acid-reactive substances according to Placer et al. (1966). The colorimetric antioxidant assay kit (Cayman Chemical Company, USA) was used for total antioxidant capacity (TAC) determination at the absorbance of 405 nm with a microplate reader. The blood serum including total protein (TP), albumin, creatinine, triglyceride, cholesterol, high-density lipoprotein cholesterol (HDL-C), low-density lipoprotein cholesterol (LDL-C), blood urea nitrogen (BUN), glucose, aspartate aminotransferase (AST), and alanine aminotransferase (ALT) were measured using an auto-analyzer (A15, Biosystem, Spain) after centrifugation (Eppendorf AG, Hamburg, Germany) at 3000×*g* for 10 min.

### Calculations and statistical analysis

The equations presented by Menke and Steingass (1988) were employed to determine net energy for lactation (NEl) and metabolism energy (ME). The data of gas production were analyzed using the following equation: $Y=b\left(1-{e}^{-ct}\right)$, where *Y* = the volume of gas production at time t, *b* = the potential gas production after 96 h incubation (mL/per 200 mg DM), *c* = the fractional rate of gas production for b (%/h), and *t* = the incubation time (h) (Ørskov and McDonald 1979).

The data related to in vitro and laboratory studies were analyzed in a completely randomized design using GLM procedure of SAS statistical package (SAS Institute Inc. 2002) with the following model: Y_ij_ = μ + T_i_ + e_ij_, where Y_ij_ = the value of each observation, μ = overall mean, T_i_ = treatment effect and e_ij_ = experimental error.

The data of in vivo section were analyzed in a randomized complete block design using GLM procedure of SAS statistical package (SAS Institute Inc. 2002) with the following model: Y_ij_ = μ + T_i_ + B_j_ + e_ij_, where Y_ij_ = the response variable, μ is the overall mean, T_i_ = the treatment effect, B_j_ = the block effect, and eij = the random error. Statistical differences between means of in vivo and in vitro data were determined using the Duncan test at *p* ≤ 0.05.

## Results

### Chemical and mineral contents

The chemical contents of some leaves are presented in Table 1. The tested leaves had different chemical content. Highest DM content (481.62 g/kg fresh weight) was obtained in quince (*p* < 0.001). The concentration of NDF ranged from 324.73 for sour cherry to 484.80 g/kg DM for olive. The content of ADF also differed from 161.20 for greengage to 299.63 g/kg DM for olive. Greengage (146.37) and persimmon (136.96 g/kg DM) exhibited the highest CP content, respectively (*p* < 0.001). Olive had the highest ADL (136.55 g/kg DM) and EE (39.67 g/kg DM) (*p* < 0.001). The highest content of NFC was observed in pear and sour cherry, respectively (*p* < 0.001). The highest concentrations for total phenol (91.49 g/kg DM) and tannins (85.76 g/kg DM) were obtained in quince (*p* < 0.001). Olive had the lowest non-tannin phenol (3.75 g/kg DM, *p* = 0.001) among tree leaves.

**Table 1:** Chemical contents (g/kg DM) of some leaves

Item	Leaf							SEM	*p*-value
	Quince	Pear	Olive	Mirabelle plum	Greengage	Sour cherry	Persimmon		
DM	481.62^a^	461.11^bc^	471.33^ab^	443.60^c^	401.18^d^	390.59^d^	353.00	3.78	< 0.001
NDF	426.67^b^	340.00^c^	484.80^a^	470.87^a^	405.97^b^	324.73^c^	478.10^a^	7.28	< 0.001
ADF	286.63^a^	189.37^bc^	299.63^a^	162.30^c^	161.20^c^	219.90^b^	208.10^bc^	9.89	< 0.001
ADL	115.64^ab^	70.34^c^	136.55^a^	74.22^c^	69.29^c^	95.12^bc^	73.62^c^	6.06	< 0.001
CP	116.50^cd^	111.38^de^	100.49^e^	127.51^bc^	146.37^a^	126.63^bc^	136.96^ab^	2.72	< 0.001
EE	18.33^ef^	17.40^f^	39.67^a^	24.00^cd^	22.00^de^	26.67^c^	33.67^b^	0.79	< 0.001
Ash	73.20^c^	76.07^c^	73.50^c^	116.20^a^	120.20^a^	91.13^b^	96.73^b^	2.73	< 0.001
NFC	365.30^b^	455.15^a^	301.54^c^	261.42^d^	305.47^c^	430.83^a^	254.53^d^	6.52	< 0.001
TP	91.49^a^	64.34^c^	61.84^cd^	62.73^c^	55.12^de^	53.22^e^	73.14^b^	2.20	< 0.001
TT	85.76^a^	56.38^cd^	58.09^c^	54.58^cd^	49.70^d^	48.68^d^	68.13^b^	2.20	< 0.001
NTP	5.72^b^	7.97^a^	3.75^c^	8.15^a^	5.42^b^	4.53^bc^	5.01^bc^	0.41	0.001

Means with different letters within the same row are significantly different according to *p*-value indicated. NFC was calculated by subtracting CP, NDF, fat, and ash contents from total DM (Sniffen et al. 1992)

*DM* (g/kg fresh weight) dry matter, *NDF* neutral detergent fiber, *ADF* acid detergent fiber, *ADL* acid detergent lignin, *CP* crude protein, *EE* ether extract, *NFC* non-fiber carbohydrates, *TP* total phenol, *TT* total tannin, *NTP* non-tannin phenol, *SEM* standard error of the mean

The mineral contents of some leaves are exhibited in Table 2. Exception cobalt, different mineral contents were observed among leaves (*p* < 0.001). Persimmon had the highest calcium (19.82 g/kg DM, *p* < 0.001). The highest contents of phosphorus (3.02 g/kg DM), potassium (19.41 g/kg DM), sodium (0.80 g/kg DM), and zinc (29.67 mg/kg DM) were obtained in mirabelle plum (*p* < 0.001). Quince had the highest concentrations of iron (136.67 mg/kg DM) and copper (11.49 mg/kg DM) (*p* < 0.001). The content of manganese (135.03 mg/kg DM) in pear was highest among leaves (*p* < 0.001). The concentration of magnesium differed from 3.73 for olive to 7.54 g/kg DM for greengage.

**Table 2:** Mineral contents of some leaves

Item	Leaf							SEM	*p*-value
	Quince	Pear	Olive	Mirabelle plum	Greengage	Sour cherry	Persimmon		
Ca	11.18^d^	15.49^bc^	17.43^b^	14.48^c^	14.59^c^	14.47^c^	19.82^a^	0.47	< 0.001
P	0.11^e^	1.65^c^	1.19^d^	3.02^a^	2.66^a^	2.13^b^	1.21^d^	0.09	< 0.001
K	9.20^d^	5.84^e^	7.33^de^	19.41^a^	16.50^b^	12.45^c^	18.37^ab^	0.51	< 0.001
Na	0.51^c^	0.38^d^	0.51^c^	0.80^a^	0.66^b^	0.52^c^	0.62^bc^	0.02	< 0.001
Mg	6.55^ab^	4.09^bc^	3.73^c^	3.96^bc^	7.54^a^	6.52^ab^	7.51^a^	0.55	< 0.001
Mn	48.10^de^	135.03^a^	49.23^de^	57.43^d^	109.13^b^	39.53^e^	75.64^c^	2.11	< 0.001
Co	2.43	2.87	2.50	2.80	2.40	2.43	2.63	0.27	0.79
Fe	136.67^a^	95.12^c^	97.57^c^	106.33^bc^	108.08^bc^	120.20^ab^	109.58^bc^	4.46	< 0.001
Zn	26.83^ab^	27.63^ab^	14.53^c^	29.67^a^	22.07^abc^	20.00^bc^	18.43^c^	1.63	< 0.001
Cu	11.49^a^	8.83^b^	3.24^d^	5.33^c^	4.83^c^	2.92^d^	3.52^d^	0.16	< 0.001

Means with different letters within the same row are significantly different according to *p*-value indicated

*Ca* calcium (g/kg DM), *P* phosphorus (g/kg DM), *K* potassium (g/kg DM), *Na* sodium (g/kg DM), *Mg* magnesium (g/kg DM), *Mn* manganese (mg/kg DM), *Co* cobalt (mg/kg DM), *Fe* iron (mg/kg DM), *Zn* zinc (mg/kg DM), *Cu* copper (mg/kg DM), *SEM* standard error of the mean

### In vitro ruminal fermentation

The pH, NH_3_-N, individual and total VFA of the culture medium following the incubation of some leaves are presented in Table 3. The highest pH of the culture medium was observed when sour cherry (6.74) and quince (6.71) were incubated, respectively (*p* < 0.001). The lowest NH_3_-N (15.67 mg/dL) was obtained in olive (*p* = 0.02). Total VFA were highest in greengage (66.07 mmol/L) and mirabelle plum (65.58 mmol/L), respectively (*p* < 0.001). Greengage (23.03), mirabelle plum (21.87), and sour cherry (20.83 mol/100 mol) exhibited the highest propionate, respectively (*p* < 0.01). Other individual VFA containing acetate, butyrate, valerate, and isovalerate were not different among tree leaves (*p* > 0.05).

**Table 3:** The pH, NH_3_-N, individual and total volatile fatty acids of the culture medium following the incubation of some leaves

Item	Leaf							SEM	*p*-value
	Quince	Pear	Olive	Mirabelle plum	Greengage	Sour cherry	Persimmon		
pH	6.71^ab^	6.65^bc^	6.67^b^	6.58^cd^	6.56^d^	6.74^a^	6.59^cd^	0.01	< 0.001
NH_3_-N (mg/dL)	16.03^ab^	16.08^ab^	15.67^b^	16.33^ab^	17.06^a^	16.46^ab^	16.79^ab^	0.25	0.02
Total VFA (mmol/L)	61.08^c^	59.75^c^	60.19^c^	65.58^ab^	66.07^a^	63.60^b^	61.43^c^	0.44	< 0.001
C2 (mol/100 mol)	62.00	61.83	62.67	62.50	62.00	63.17	62.17	0.49	0.53
C3 (mol/100 mol)	19.83^bc^	19.33^c^	20.77^abc^	21.87^ab^	23.03^a^	20.83^abc^	20.07^bc^	0.51	< 0.01
C4 (mol/100 mol)	13.67	13.53	13.00	13.07	12.87	13.00	13.90	0.44	0.57
C5 (mol/100 mol)	1.28	1.47	1.38	1.39	1.25	1.30	1.50	0.08	0.29
Iso-C5 (mol/100 mol)	0.42	0.47	0.41	0.48	0.41	0.44	0.51	0.04	0.56

Means with different letters within the same row are significantly different according to *p*-value indicated

*NH*_*3*_*-N* ammonia nitrogen, *VFA* volatile fatty acids, *C2* acetate, *C3* propionate, *C4* butyrate, *C5* valerate, *Iso-C5* isovalerate, *SEM* standard error of the mean

The gas test parameters, ME, NEl, true nutrient digestibility, microbial mass yield (MMY), and PF obtained for some leaves are presented in Table 4. Different gas production parameters were observed among tree leaves. The highest amount of 24 h gas production was observed in greengage (48.30) and mirabelle plum (44.83 mL), respectively (*p* < 0.001). The lowest potential gas production was related to pear leaves (39.65 mL, *p* < 0.001). Persimmon (0.054), quince (0.055), and olive (0.057%/h) had the lowest fractional rate of gas production compared to other leaves, respectively (*p* < 0.001). Greengage had the highest content of ME (9.73 MJ/kg DM) and NEl (5.81 MJ/kg DM) (*p* < 0.001). The content of TDMD ranged from 644.91 for pear to 824.56 g/kg DM for greengage (*p* < 0.001). Greengage (831.50), mirabelle plum (820.67), and sour cherry (801.17 g/kg DM) had the highest TOMD, respectively (*p* < 0.001). The microbial mass yield was different from 37.33 for pear to 63.27 mg for greengage, respectively (*p* < 0.001). The lowest PF was obtained in pear (3.02), persimmon (3.29), and olive (3.33), respectively (*p* < 0.001).

**Table 4:** The gas test parameters, ME, NEl, true nutrient digestibility, MMY, and PF measured for some leaves

Item	Leaf							SEM	*p*-value
	Quince	Pear	Olive	Mirabelle plum	Greengage	Sour cherry	Persimmon		
gas 12 h (mL)	27.06^b^	27.20^b^	26.10^bc^	35.23^a^	36.16^a^	32.16^a^	21.93^c^	1.03	< 0.001
gas 24 h (mL)	36.70^b^	33.60^b^	34.63^b^	44.83^a^	48.30^a^	37.13^b^	36.73^b^	0.86	< 0.001
gas 48 h (mL)	47.50^bc^	37.90^d^	47.30^bc^	52.77^a^	51.30^ab^	44.83^c^	44.93^c^	0.96	< 0.001
gas 72 h (mL)	51.60^ab^	40.80^c^	51.13^b^	55.67^a^	52.53^ab^	48.50^b^	50.47^b^	0.93	< 0.001
gas 96 h (mL)	53.03^abc^	42.03^d^	52.50^abc^	57.20^a^	55.20^ab^	49.80^c^	51.36^bc^	1.07	< 0.001
b (mL)	52.36^ab^	39.65^d^	51.48^abc^	55.32^a^	52.18^ab^	47.29^c^	50.69^bc^	0.94	< 0.001
c (%/h)	0.055^d^	0.104^b^	0.057^d^	0.081^c^	0.125^a^	0.088^c^	0.054^d^	0.003	< 0.001
ME (MJ/kg DM)	7.94^c^	7.48^d^	7.92^c^	9.17^b^	9.73^a^	8.16^c^	8.29^c^	0.12	< 0.001
NEl (MJ/kg DM)	4.56^c^	4.24^d^	4.56^c^	5.42^b^	5.81^a^	4.70^c^	4.78^c^	0.09	< 0.001
TDMD (g/kg DM)	679.18^c^	644.91^c^	666.65^c^	808.25^ab^	824.56^a^	786.77^b^	657.03^c^	7.37	< 0.001
TOMD (g/kg DM)	690.33^b^	660.73^b^	689.67^b^	820.67^a^	831.50^a^	801.17^a^	684.83^b^	8.27	< 0.001
MMY (mg)	38.50^c^	37.33^c^	38.10^c^	61.80^a^	63.27^a^	49.17^b^	38.33^c^	1.49	< 0.001
PF	3.53b^c^	3.02^d^	3.33^cd^	3.87^ab^	4.15^a^	3.91^ab^	3.29^cd^	0.08	< 0.001

Means with different letters within the same row are significantly different according to *p*-value indicated

*gas 12, 24, 48, 72, and 96 h* cumulative gas production after 12, 24, 48, 72, and 96 h incubation; *b* potential gas production; *c* fractional rate of gas production

*ME* metabolism energy, *NEl* net energy for lactation, *TDMD* true dry matter digestibility, *TOMD* true organic matter digestibility, *MMY* microbial mass yield, *PF* partitioning factor (mg TOMD/mL 24 h gas production), *SEM* standard error of the mean

### Buffering capacity parameters

The pH, titratable acidity, acid-buffering capacity, titratable alkalinity, base-buffering capacity, and acid–base buffering capacity (mEq × 10^–3^) of some leaves are shown in Table 5. The value of pH (5.26) for mirabelle plum was lowest among tree leaves (*p* < 0.001). Sour cherry had the highest titratable acidity (258.75 mEq × 10^–3^) and acid–base buffering capacity (183.73 mEq × 10^–3^) (*p* < 0.001). Also, sour cherry (113.13) and mirabelle plum (108.17 mEq × 10^–3^) exhibited the highest acid buffering capacity, respectively (*p* < 0.001). The highest amounts of titratable alkalinity (271.25 mEq × 10^–3^) and base-buffering capacity (84.70 mEq × 10^–3^) were observed in quince (*p* < 0.001).

**Table 5:** The pH, titratable acidity, acid-buffering capacity, titratable alkalinity, base-buffering capacity, and acid–base buffering capacity (mEq × 10^–3^) of some leaves

Item	Leaf							SEM	*p*-value
	Quince	Pear	Olive	Mirabelle plum	Greengage	Sour cherry	Persimmon		
pH	6.03^b^	5.92^c^	5.87^c^	5.26^f^	5.71^d^	6.29^a^	5.57^e^	0.02	< 0.001
Titratable acidity	172.75^b^	164.75^b^	148.00^c^	136.75^d^	152.25^c^	258.75^a^	127.25^d^	2.25	< 0.001
Acid-buffering capacity	85.13^bcd^	85.69^bc^	79.31^d^	108.17^a^	88.77^b^	113.13^a^	81.05^cd^	1.30	< 0.001
Titratable alkalinity	271.25^a^	228.50^cd^	215.50^de^	241.75^bc^	247.50^b^	204.75^e^	231.25^bcd^	4.11	< 0.001
Base-buffering capacity	84.70^a^	68.36^bc^	63.76^cd^	60.48^d^	67.99^bc^	70.60^b^	63.48^cd^	1.17	< 0.001
Acid–base buffering capacity	169.83^b^	154.05^cd^	143.07^e^	168.65^b^	156.77^c^	183.73^a^	144.54^de^	2.24	< 0.001

Means with different letters within the same row are significantly different according to *p*-value indicated

*SEM* standard error of the mean

### Animal experiment

#### Performance and nutrient digestibility

Ingredients and chemical composition of diets containing different leaves are shown in Table 6. The diets were relatively considered as isonitrogenous and isoenergetic. The effects of diets containing different leaves on nutrient digestibility and goats’ performance are presented in Table 7. The highest dry matter intake (DMI, 1087 g/day) was related to diets containing greengage leaves (*p* < 0.001). Initial body weight (BW) was unchanged at the start of experiment (*p* > 0.05), but final BW (*p* = 0.001) and average daily gain (ADG, *p* = 0.05) were highest for diets containing greengage and mirabelle plum, respectively. Different nutrient digestibility was observed among treatments (*p* < 0.001). Greengage had the highest nutrient digestibility (exception EE) compared to the control group (*p* < 0.001).

**Table 6:** Ingredients and chemical composition of diets containing different leaves

Item	Treatment							
	Control	Quince	Pear	Olive	Mirabelle plum	Greengage	Sour cherry	Persimmon
Ingredients (% of DM)								
Alfalfa hay	27	13.5	13.5	13.5	13.5	13.5	13.5	13.5
Corn silage	13	6.5	6.5	6.5	6.5	6.5	6.5	6.5
Tree leaf	0	20	20	20	20	20	20	20
Barley grain	18	18	18	18	18	18	18	18
Corn grain	18	18	18	18	18	18	18	18
Canola meal	9.5	9.5	9.5	9.5	9.5	9.5	9.5	9.5
Wheat bran	9.5	9.5	9.5	9.5	9.5	9.5	9.5	9.5
Vitamine-mineral^a^	2	2	2	2	2	2	2	2
Magnesium oxide	1	1	1	1	1	1	1	1
Dicalcium phosphate	1	1	1	1	1	1	1	1
Salt	1	1	1	1	1	1	1	1
Chemical composition								
DM (% of fresh weight)	67.32	76.95	76.95	76.95	76.95	76.95	76.95	76.95
CP (% of DM)	14.59	14.44	14.34	14.32	14.66	14.94	14.64	14.85
NDF (% of DM)	35.95	32.76	31.03	33.93	33.65	32.35	30.72	33.79
NFC (% of DM)	39.5	42.7	44.4	41.3	41.5	41.6	44	40.6
Ash (% of DM)	10.4	10.2	10.26	10.21	10.21	11.14	10.56	10.67
EE (% of DM)	2.67	2.66	2.64	3.09	2.77	2.73	2.83	2.97
ME (Mcal/kg DM)	2.28	2.30	2.31	2.27	2.28	2.27	2.30	2.27
TP (% of DM)	0	1.83	1.29	1.24	1.26	1.10	1.06	1.46
TT (% of DM)	0	1.72	1.13	1.16	1.09	0.99	0.97	1.36
NTP (% of DM)	0	0.11	0.16	0.08	0.16	0.11	0.09	0.10

*Control* basal diet containing alfalfa hay and corn silage, *DM* dry matter, *CP* crude protein, *NDF* neutral detergent fiber, *NFC* non-fiber carbohydrates, *EE* ether extract, *ME* metabolism energy, *TP* total phenol, *TT* total tannin, *NTP* non-tannin phenol

^a^Per kg of premix contained 100 mg vitamin E, 10 mg vitamin B1, 20 mg vitamin B2, 400,000 IU vitamin A, 100,000 IU vitamin D, 3% calcium, 1.2% phosphorus, 4% sodium, 1.1% magnesium, 1000 mg copper, 60 mg iodine, 2000 mg manganese, 2000 mg zinc, 3000 mg iron, 60 mg cobalt

**Table 7:** The effects of diets containing different leaves on nutrient digestibility and growth performance of goats

Item	Treatment								SEM	*p*-value
	Control	Quince	Pear	Olive	Mirabelle plum	Greengage	Sour cherry	Persimmon		
DMI (g/day)	979^d^	977^d^	973^d^	971^d^	1045^b^	1087^a^	1003^c^	0.973^d^	0.008	< 0.001
Initial BW (kg)	19.14	19.10	19.21	19.21	19.38	19.19	19.06	19.09	0.69	0.97
Final BW (kg)	28.42^bc^	28.70^bc^	27.21^c^	28.06^c^	29.69^ab^	30.55^a^	28.75^bc^	28.37^bc^	0.48	0.001
ADG (kg/day)	0.114^bc^	0.120^abc^	0.100^c^	0.111^bc^	0.129^ab^	0.142^a^	0.121^abc^	0.116^abc^	0.009	0.05
Nutrient digestibility (%)										
DM	64.33^bc^	64.11^ cd^	63.72^ cd^	63.39^d^	65.04^ab^	65.42^a^	65.06^ab^	64.40^bc^	0.28	< 0.001
NDF	40.90^bc^	39.00^d^	41.30^abc^	39.14^d^	42.16^ab^	42.57^a^	42.02^abc^	40.48^cd^	0.52	< 0.001
CP	67.51^bc^	67.48^bc^	66.36^ cd^	65.70^d^	68.93^b^	70.80^a^	68.61^b^	67.36^bc^	0.53	< 0.001
OM	67.16^ cd^	67.10^ cd^	66.06^d^	66.11^d^	68.86^ab^	70.12^a^	68.39^bc^	66.94^cd^	0.47	< 0.001
EE	50.42^ fg^	49.91^ g^	52.90^ cd^	55.46^a^	52.08^de^	51.43^ef^	53.86^bc^	54.88^ab^	0.49	< 0.001

Means with different letters within the same row are significantly different according to *p*-value indicated

*Control* basal diet containing alfalfa hay and corn silage, *DMI* dry matter intake, *BW *body weight, *ADG* average daily gain, *DM* dry matter, *NDF* neutral detergent fiber, *CP* crude protein, *OM* organic matter, *EE* ether extract, *SEM* standard error of the mean

#### Blood plasma and hematology parameters

The effects of diets containing different leaves on plasma and hematology parameters of goats are presented in Table 8. There was a significant difference for some plasma parameters among animals fed diets containing different leaves, but hematology parameters were unchanged. Goats fed on diets containing persimmon and quince exhibited the lowest triglyceride (*p* = 0.02), cholesterol (*p* < 0.001) and LDL-C (*p* < 0.001) and highest HDL-C (*p* < 0.001) compared to the control group. Rather than control group, greengage had the highest BUN (*p* = 0.05) and the lowest glucose concentration was observed in olive (*p* < 0.001). The concentrations of AST and ALT in quince were significantly decreased compared to the control group (*p* = 0.05). Olive, persimmon, and quince had the highest TAC and lowest MDA rather than other treatments (*p* < 0.001).

**Table 8:** The effects of diets containing different leaves on plasma and hematology parameters of goats

Item	Treatment								SEM	*p*-value
	Control	Quince	Pear	Olive	Mirabelle Plum	Greengage	Sour cherry	Persimmon		
Plasma parameters										
Total protein (g/dL)	6.85	6.87	6.90	6.98	7.00	7.09	6.96	6.90	0.08	0.50
Albumin (g/dL)	3.56	3.53	3.61	3.47	3.65	3.71	3.61	3.61	0.12	0.92
Creatinine (mg/dL)	0.92	0.93	0.94	1.00	0.89	0.92	1.00	0.93	0.04	0.41
Triglyceride (mg/dL)	36.38^a^	34.53^b^	35.79^ab^	35.98^ab^	36.77^a^	36.54^a^	36.35^a^	34.41^b^	0.53	0.02
Cholesterol (mg/dL)	67.91^a^	64.82^b^	67.70^a^	67.16^a^	67.71^a^	68.43^a^	67.48^a^	64.04^b^	0.67	< 0.001
HDL-C (mg/dL)	43.07^b^	45.98^a^	43.88^b^	43.72^b^	43.90^b^	43.32^b^	44.40^b^	46.45^a^	0.44	< 0.001
LDL-C (mg/dL)	18.75^a^	15.37^b^	18.75^a^	18.58^a^	18.46^a^	18.66^a^	18.03^a^	16.12^b^	0.40	< 0.001
BUN (mg/dL)	15.95^b^	15.77^b^	15.71^b^	15.89^b^	16.07^ab^	16.96^a^	16.33^ab^	15.59^b^	0.30	0.05
Glucose (g/dL)	63.51^a^	62.68^a^	62.22^a^	59.70^b^	64.09^a^	64.10^a^	64.11^a^	62.78^a^	0.41	< 0.001
AST (U/L)	48.54^a^	46.89^b^	49.09^a^	49.40^a^	49.10^a^	49.10^a^	48.83^a^	48.82^a^	0.55	0.05
ALT (U/L)	17.07^a^	15.64^b^	16.86^a^	16.97^a^	17.00^a^	16.84^a^	16.91^a^	16.77^a^	0.30	0.05
TAC (mmol/L)	0.91^c^	1.13^a^	0.90^c^	1.16^a^	0.88^c^	0.89^c^	1.02^b^	1.12^a^	0.03	< 0.001
MDA (nmol/mL)	2.73^a^	2.32^c^	2.84^a^	2.33^c^	2.84^a^	2.84^a^	2.50^b^	2.29^c^	0.06	< 0.001
Hematology										
WBC (Cell/μL)	10,383.3	10,770.3	10,833.7	10,580.3	10,651.2	10,741.0	10,816.7	10,832.3	206.45	0.77
RBC (× 10^6^/μL)	15.53	15.05	15.07	15.03	15.56	15.40	15.69	15.66	0.29	0.51
MCV (fL)	17.95	17.34	17.61	17.37	17.94	17.32	18.25	18.04	0.34	0.36
MCH (pg)	6.12	6.08	6.14	6.12	6.13	6.07	6.20	6.17	0.11	0.98
MCHC (g/dL)	33.84	33.51	33.40	33.08	33.36	33.29	33.89	33.58	0.42	0.88
PCV (%)	27.37	27.25	27.26	27.29	28.45	28.15	28.54	28.57	0.49	0.17
Hemoglobin (g/dL)	9.42	9.37	9.59	9.54	9.53	9.38	9.86	9.67	0.19	0.64

Means with different letters within the same row are significantly different according to *p*-value indicated

*Control* basal diet containing alfalfa hay and corn silage, *HDL-C* high-density lipoprotein cholesterol, *LDL-C* low-density lipoprotein cholesterol, *BUN* blood urea nitrogen, *AST* aspartate aminotransferase, *ALT* alanine aminotransferase, *TAC* total antioxidant capacity, *MDA* malondialdehyde, *WBC* white blood cell, *RBC* red blood cell, *MCV* mean corpuscular volume, *MCH* mean corpuscular hemoglobin, *MCHC* mean corpuscular hemoglobin concentration, *PCV* packed cell volume, *SEM* standard error of the mean

## Discussion

### Chemical and mineral contents

In line with the present study, different chemical composition was reported for some tree leaves (*Prosopis cineraria*, *Acacia nilotica*, and *Albezia lebbek*) (Bhatta et al. 2005). Moderate to high levels of CP in the studied leaves confirm their high nutritive value. It is reported that tree leaves contain high levels of CP and they are valuable for ruminants fed low-quality fodders, especially during the dry season when it is not possible to provide high-quality forages (Salem et al. 2007). Topps (1992) reported that tree fodders are richer in CP, minerals, and digestible nutrients compared to grasses. In line with the present study, phenolic and tannin compounds have been reported for some tree leaves containing *Acacia cyanophylla* Lindl. (Ben Salem et al. 2005), *Prosopis cineraria*, *Acacia nilotica*, and *Albezia lebbek* (Bhatta et al. 2005). Tannins are a group of phenolic compounds that are naturally present in plants and their normal consumption can be beneficial to livestock (Dawson et al. 1999). The effects of these compounds are different but high consumption is often not recommended due to the binding of dietary carbohydrates and proteins and subsequently decrease in carbohydrates and protein digestibility (Martens et al. 2013). In the present study, a diverse range of tannins (48.68–85.76 g/kg DM) was observed among tree leaves. Although the inclusion of diets with phenolic compounds because of anti-inflammatory and antimicrobial activities along with antioxidant capacity can have beneficial effects on animal performance (Ignat et al. 2011), the suitable levels of phenolic compounds (53.22–91.49 g/kg DM) was observed among the studied leaves. A lower concentration of NTP (3.7–8.1 g/kg DM) for the studied leaves was observed than those reported for other tree leaves (10–57 g/kg DM) including *Sesbania grandiflora*, *Melia dubia*, *Dillenia* spp., *Artocarpus heterophyllus*, *Commiphora caudata*, *Moringa oleifera*, *Leucaena leucocephala*, and *Acacia auriculiformis* (Giridhar et al. 2018).

Determining the mineral composition of studied leaves can be useful in preparing a balanced diet for small ruminants. Also, animal feed is one of the major sources of minerals and utilization of agro-industrial by-products may also affect the mineral status of livestock (Ribeiro et al. 2019). In the present study, concentration of calcium reported for all leaves was higher than those (1.4–7.0 g/Kg DM) reported for fodders (Freer et al. 2007). The contents of phosphorus (exception quince), potassium, magnesium, and sodium (exception pear, quince, olive, persimmon, and sour cherry) of studied leaves were respectively within or above the recommended dietary levels of 0.9–3, 5, 0.9–1.2, and 0.7–1 g/kg DM for sheep (Freer et al. 2007). The mineral requirements of goats for copper, cobalt, manganese, and zinc are reported about 8–10, 0.1, 40–50, and 50 mg/Kg DM of diet, respectively (Meschy 2000). In this regard, pear (8.83) and quince (11.49 mg/kg DM) leaves can easily meet the copper requirements of goats. Also, the concentrations of cobalt (2.40–2.87 mg/kg DM) and manganese (39.53–135.03 mg/kg DM) in the studied leaves are adequate in goat feeding, but they are deficient in zinc (14.53–29.67 mg/kg DM). The iron content of all leaves was above the recommended dietary levels (30–50 mg/kg DM) for sheep (Moniello et al. 2005).

#### In vitro ruminal fermentation

In the present study, similar to report of Getachew et al. (2004), a different molar proportions of VFA in the culture medium was observed. A decrease in pH value of the culture medium following the inclusion of greengage could be attributed to higher VFA yield compared to other leaves (Dijkstra et al. 2012). As Kazemi (2019) reported a positive correlation between CP and ammonia nitrogen of culture medium, a lower NH_3_-N in olive leaves can be related to its more CP.

The higher 24 h gas production in greengage and mirabelle plum can be attributed to higher total VFA produced in the culture medium as Kazemi and Valizadeh (2019) stated that a strong positive correlation has been observed between total VFA and gas production. Close to the present results, Shakeri et al. (2017) reported that the volume of gas produced after 96 h incubation for two varieties of olive leaves was 49.40 and 49 mL/200 mg DM, respectively. Differences in gas production among the leaves of the present study can be attributed to various chemical compositions (e.g. proportion, and nature of fiber) (Rubanza et al. 2003) along with different tannin content that may negatively affect the activity of rumen microorganisms (Goel and Makkar 2012). Despite greater content of tannin in quince leaves rather than other leaves, lowered potential gas production in pear leaves confirmed different effects of tannin nature besides its concentration (Makkar 2003a). In contrast, quince leaves (high level of tannin) resulted in a lower fractional rate of gas production (cgas). It is reported that tannins can reduce the rate of digestion to a greater extent than the potential extent of digestion (Makkar et al. 1995). In this regard, TDMD of leaves with lower tannin concentration (sour cherry, greengage, and mirabelle plum) was higher than leaves (quince and persimmon) containing higher tannin. In contrast with the present findings, some tropical tannin‐containing leaves such as *Syzygium cumini*, *Azadirachta indica*, *Ficus religiosa*, and *Acacia nilotica* exhibited greater OM degradability and they improved microbial biomass yield (Pal et al. 2015). The higher PF in greengage leaves indicated higher efficiency of microbial protein synthesis compared to other leaves. As PF is considered as an index of distribution of TOMD between fermentation gases and microbial biomass, and increased PF means less gas and more microbial biomass (Pawar et al. 2014), therefore higher PF in greengage leaves might be useful in reducing environmental pollution.

The buffering capacity of some protein sources and leguminous fodder has been reported to be greater than 85 mEq × 10^–3^ (Montañez-Valdez et al. 2013), which is consistent with the present study. The greatest acid–base buffering capacity in sour cherry leaves (183.73 mEq × 10^–3^) represented high control of it in maintaining the pH balance. Also, the highest acid buffering capacity was obtained in sour cherry leaves (113.13 mEq × 10^–3^), indicating that more acid is needed to change the pH of the water-soluble sample. It is reported that initial pH and titratable acidity are two major factor affecting on ruminal pH. In the present study, the highest titratable acidity was obtained for sour cherry (258.75 mEq × 10^–3^), indicating high resistance to acidification. Given the value of pH and buffering capacity of the diets, it would be possible to predict the need for buffers to control and maintain rumen pH (Bujňák et al. 2011). All leaves had near-neutral pH and therefore their consumption may not lead to changes in rumen pH. Also, the amount and compound of minerals in crude ash have a particular buffering effect on the plant's initial pH (Lević et al. 2005). Due to the different ash content of the studied leaves (73.20–120.20 g/kg DM), their buffering capacity was also different.

### Goat experiments

This is the first experiment to evaluate some tree leaves in vivo. So, we had to compare the present results with other tree leaves containing tannin or phenolic contents. Feeding of all studied tree leaves to goats had no negative effect on DMI compared to the control group which may be due to better palatability (Raghuvansi et al. 2007). Although diets of present study were relatively isonitrogenous and isoenergetic, final BW and ADG increased in goats fed the greengage diet compared to the control group. This improvement could be related to an increase in DMI and nutrient digestibility (Table 7), and an increase in propionate concentration of culture medium following the incubation of greengage (Table 3). In line with the present results, goats fed on *Paraserianthes falcataria*, *Moringa oleifera*, and *Calliandra calothyrsus* leaves exhibited higher growth performance, nutrient digestibility, and feed efficiency when were compared to those fed on grass hay (Okoruwa and Ikhimioya 2020). The positive and negative effects of tannins have been reported by Makkar (2003a). It is reported that the great concentration of tannins in leaves can have deleterious effects on nutrient digestibility and nitrogen retention (Kamalak 2006). The use of some browse leaves is restricted because of their great tannin contents (Silanikove et al. 2001). Barry et al. (1986) reported that less than 4% of tannin in the diet can be beneficial for ruminants. In this regard, the concentration of tannins in the diets of the present study ranged from 0 to 1.72% DM. So, no deleterious effect of these amounts of tannins on the performance of tree leaves-fed goats was not observed.

The concentration of blood metabolites is an indicator for assessing the content of nutrients in the diet, and they will affect the physiological conditions of animals (Pambu-Gollah et al. 2000). Differences in some blood metabolites among the treatments were probably due to different DMI, nutrient digestibility, and ruminal fermentation parameters. The lower triglyceride, cholesterol, LDL-C, and higher HDL-C in goats fed on diets containing quince and persimmon leaves can be attributed to the lipid-lowering properties of quince (Umar et al. 2015) and persimmon (Zhang et al. 2016) as a result of high total flavonoids contents. A higher BUN concentration was observed in diet containing greengage compared to the control group. This increase in BUN can be related to increasing in CP digestibility and DMI. The concentration of BUN may be useful as an index of protein status among animals, and could help to prepare the nitrogen-balanced diets or identify problems with a feeding program (Kohn et al. 2005). In line with the present results, a linear decline in plasma glucose of kids was observed when alfalfa hay was substituted with olive leaves at levels of 7.5 and 15% of diet (Jabalbarezi Hukerdi et al. 2019). Previous studies demonstrated that olive (leaf or mill waste) extracts, or its main phenolic compound (oleuropein) has anti-diabetic effects (Liu et al. 2014; Guex et al. 2019). A decrease in serum AST and ALT of goats fed a diet containing quince leaves compared to the control group is in line with Adiban et al. (2019) who demonstrated quince had a preventive effect against diethylnitrosamine-induced liver cancer by decreasing serum biomarkers (ALT and AST) responsible for liver health. An increased TAC and a declined MDA in diets containing olive, persimmon, and quince leaves can be related to their antioxidant effects (Umar et al. 2015; Xie et al. 2015; Jabalbarezi Hukerdi et al. 2019). Indeed MDA is an output of the oxidation of unsaturated fatty acid induced by free radicals which its concentration could indirectly reflect the rate of body fat oxidation (Liu and Ng 2000). In this study, lack of significant effects on the hematological parameters of goats indicates there were no negative effects on animal health.

The data presented (laboratory, in vivo and in vitro) here confirm that all tree leaves have nutritional potential and some of dietary forage can be substituted with these leaves in the dry season, especially when availability of conventional forages are scarce. In terms of DMI, final BW, ADG, and nutrient digestibility (DM, OM, NDF, and CP), greengage leaves are preferred than other species. Quince and persimmon leaves had advantages in blood lipids lowering. The amounts of serum TAC and MDA improved when adding olive, persimmon, and quince leaves to goat’s diets. Generally, each of the studied leaves had the advantages in goats feeding. However, more research and longer studies should be conducted to validate the positive and negative effects of supplementing these leaves in finishing goats and to determine the moderate feeding rates in dairy goats or other animals.

## Data Availability

Not applicable.

## Abbreviations

DM: Dry matter
CP: Crude protein
ADF: Acid detergent fiber
NDF: Neutral detergent fiber
ADL: Acid detergent lignin
EE: Ether extract
NTP: Non-tannin phenolic
EDTA: Ethylenediaminetetraacetic acid
DMI: Dry matter intake
OM: Organic matter
BW: Body weight
TAC: Total antioxidant capacity
ADG: Average daily gain
MDA: Malondialdehyde
RBC: Red blood cells
WBC: White blood cells
VFA: Volatile fatty acids
AST: Aspartate aminotransferase
ALT: Alanine aminotransferase
HDL-C: High-density lipoprotein cholesterol
LDL-C: Low-density lipoprotein cholesterol
TP: Total protein
Hb: Hemoglobin
MCV: Mean corpuscular volume
MCH: Mean corpuscular hemoglobin
MCHC: Mean corpuscular hemoglobin concentration
PCV: Packed cell volume
NEl: Net energy for lactation
ME: Metabolism energy
TDMD: True dry matter digestibility
TOMD: True organic matter digestibility
MMY: Microbial mass yield
